# Three new species of *Talaromyces* sect. *Talaromyces* discovered from soil in China

**DOI:** 10.1038/s41598-018-23370-x

**Published:** 2018-03-21

**Authors:** Xian-Zhi Jiang, Zhong-Dong Yu, Yong-Ming Ruan, Long Wang

**Affiliations:** 1 Novozymes (China) Investment Co. Ltd, Beijing, 100085 China; 2 College of Forestry, Northwest A&F University, Yangling, 712100 Shaanxi China; 3 College of Chemistry and Life Sciences, Zhejiang Normal University, Jinhua, 321004 Zhejiang, China; 4 State Key Laboratory of Mycology, Institute of Microbiology, Chinese Academy of Sciences, Beijing, 100101 China

## Abstract

Three new *Talaromyces* species isolated from soil are reported here, namely *T*. *dimorphus* (ex-type strain AS3.15692 ^T^), *T*. *lentulus* (ex-type strain AS3.15689 ^T^) and *T*. *mae* (ex-type strain AS3.15690 ^T^). *T*. *dimorphus* is characterized by biverticillate and monoverticillate penicilli, ampulliform phialides, slimy texture with sparse mycelial funicles and absent conidiogenesis on MEA. *T*. *lentulus* is featured by vivid yellow mycelium on Cz and MEA, absent conidiogenesis on CYA, and globose smooth-walled conidia. *T*. *mae* presents sparse conidia on CYA and YES, funiculous and floccose texture on MEA, and ovoid smooth-walled conidia. Both morphological and molecular characters show that *T*. *dimorphus* is unique and has no close relatives. Although *T*. *lentulus* and *T*. *mae* resembles *T*. *adpressus* and *T*. *pinophilus* very much, phylogenetic analyses of *CaM*, *BenA*, *ITS* and *Rpb2* sequences all support their status as novel species.

## Introduction

The genus *Talaromyces* was established by Benjamin^1^ in year 1955 to include the species of certain penicillia producing teleomorphic gymnothecial ascocarps with asci borne in short chains or singly, anamorphic symmetrical biverticillate penicilli, and vivid yellow, orange or pink mycelium, which belong to *Penicillium* section *Biverticillata-Symmetrica* series *Penicillium luteum* according to Raper and Thom^2^. Stolk and Samson^3^ proposed a new genus, i. e. *Hamigera* to accommodate species with asci borne singly from crosiers, and left those whose asci borne in chains to *Talaromyces*. Pitt^4^ dealt with the teleomorphic and anamorphic species in different ways owing to the dual nomenclature, regarding the teleomorphic species of this group of moulds as *Talaromyces* and anamorphic ones as *Penicillium* subgenus *Biverticillium*, respectively. In the year of 2012, the dual naming system was repealed, using a single name for a single species instead^5^. Then, the genus *Talaromyces* consists of those species showing the above-mentioned characters, regardless of sexual or asexual states.

In the study of Samson *et al*.^6^, 71 species were listed in the genus *Talaromyces*. Houbraken *et al*.^7^ established a new genus, *Rasamsonia* to accommodate the thermotolerant and thermophilic species from *Talaromyces* and *Geosmithia*, so the genus *Talaromyces* only contains mesophilic species in Trichocomaceae. In the following years, many new taxa of *Talaromyces* were reported. For example, Visagie and Jacobs^8^ discovered 3 new species, Manoch *et al*.^9^ 2 new species, Peterson and Jurjević^10^ 1 new member, Sang *et al*.^11^ 2 new taxa, Frisvad *et al*.^12^ 1 new species. In the monographic work of 2014, Yilmaz *et al*.^13^ accepted 88 species and divided *Talaromyces* into 7 sections, namely sections *Talaromyces*, *Helici*, *Purpurei*, *Trachyspermi*, *Bacillispori*, *Subinflati* and *Islandici*. In the coming few years, Visagie *et al*.^14^ reported 5 new members of section *Talaromyces*. Yilmaz *et al*.^15^ found 4 new members of section *Islandici*. Wang *et al*.^16^ added 2 new ones to section *Talaromyces*. Romero *et al*.^17^ and Luo *et al*.^18^ each reported 1 novelty of section *Trachyspermi*. Wang *et al*.^19^ discovered 1 new species of section *Talaromyces*. Yilmaz *et al*.^20^ reported 3 new species of section *Talaromyces*, and 1 of section *Bacillispori*. Chen *et al*.^21^ discovered 9 new species, among which, 3 in section *Talaromyces*, 2 in section *Helici*, 3 in section *Islandici* and 1 in section *Trachyspermi*. Crous *et al*.^22^ discovered 1 novel species belonging to section *Talaromyces*. Wang *et al*.^23^ reported 2 species belonging to section *Trachyspermi* and section *Talaromyces* respectively. Guevara-Suarez *et al*.^24^ added 2 novelties to section *Talaromyces*, 1 to section *Helici* and 1 to section *Trachyspermi*. Peterson and Jurjević^25^ dicovered 11 novelties, among them 10 belong to sect. *Islandici* and 1 to sect. *Subinflata*.

In the survey of moulds around China, many isolates belonging to *Talaromyces* were discovered. Here, we report 3 new taxa of section *Talaromyces*, namely *T*. *dimorphus* sp. nov., *T*. *lentulus* sp. nov. and *T*. *mae* sp. nov.

## Results

PCR amplification gave amplicons of *CaM* about 650 bp, *BenA* about 410 bp, *ITS* about 560 bp and *Rpb2* about 850 bp. The trimmed alignments of *CaM*, *BenA*, *ITS*, *Rpb2* and the combined *CaM*-*BenA-ITS* sequences were 494, 373, 453, 714 and 1329 characters with gaps, respectively.

**Figure 1:**
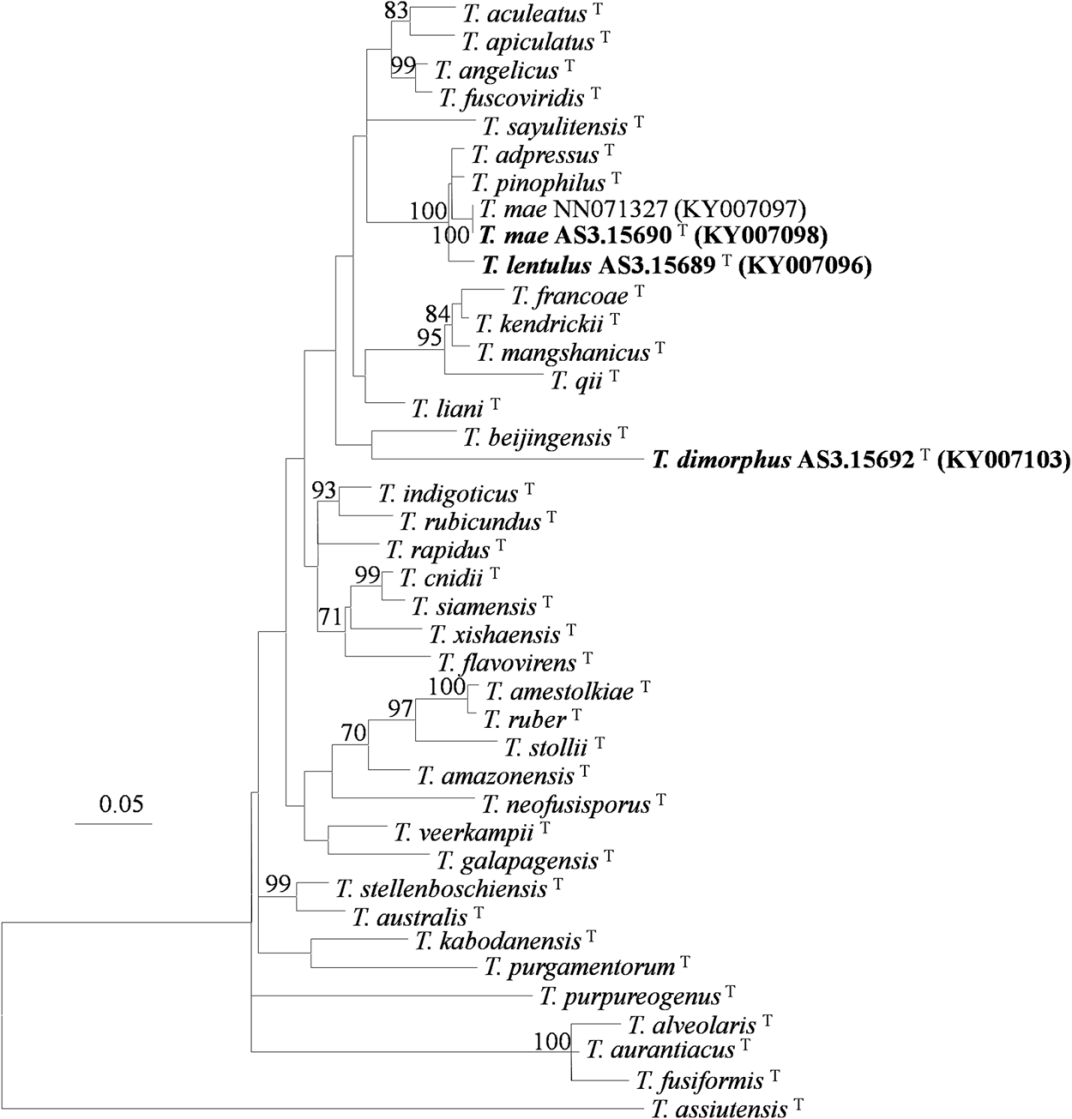
ML phylogram inferred from partial *CaM* sequences. Bootstrap percentages over 70% derived from 1000 replicates are indicated at the nodes. Bar = 0.05 substitutions per nucleotide position.

**Figure 2:**
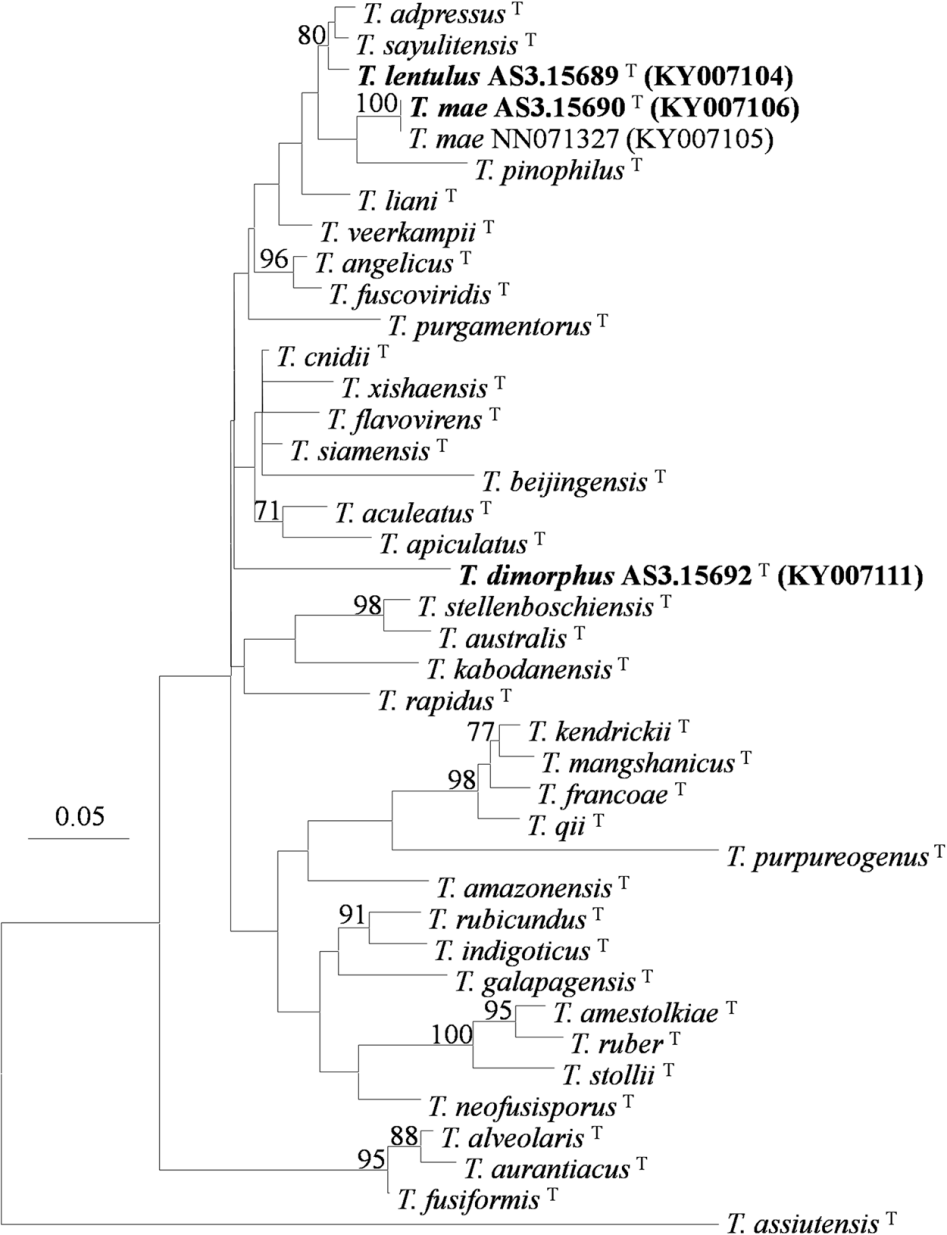
ML phylogram inferred from partial *BenA* sequences. Bootstrap percentages over 70% derived from 1000 replicates are indicated at the nodes. Bar = 0.05 substitutions per nucleotide position.

**Figure 3:**
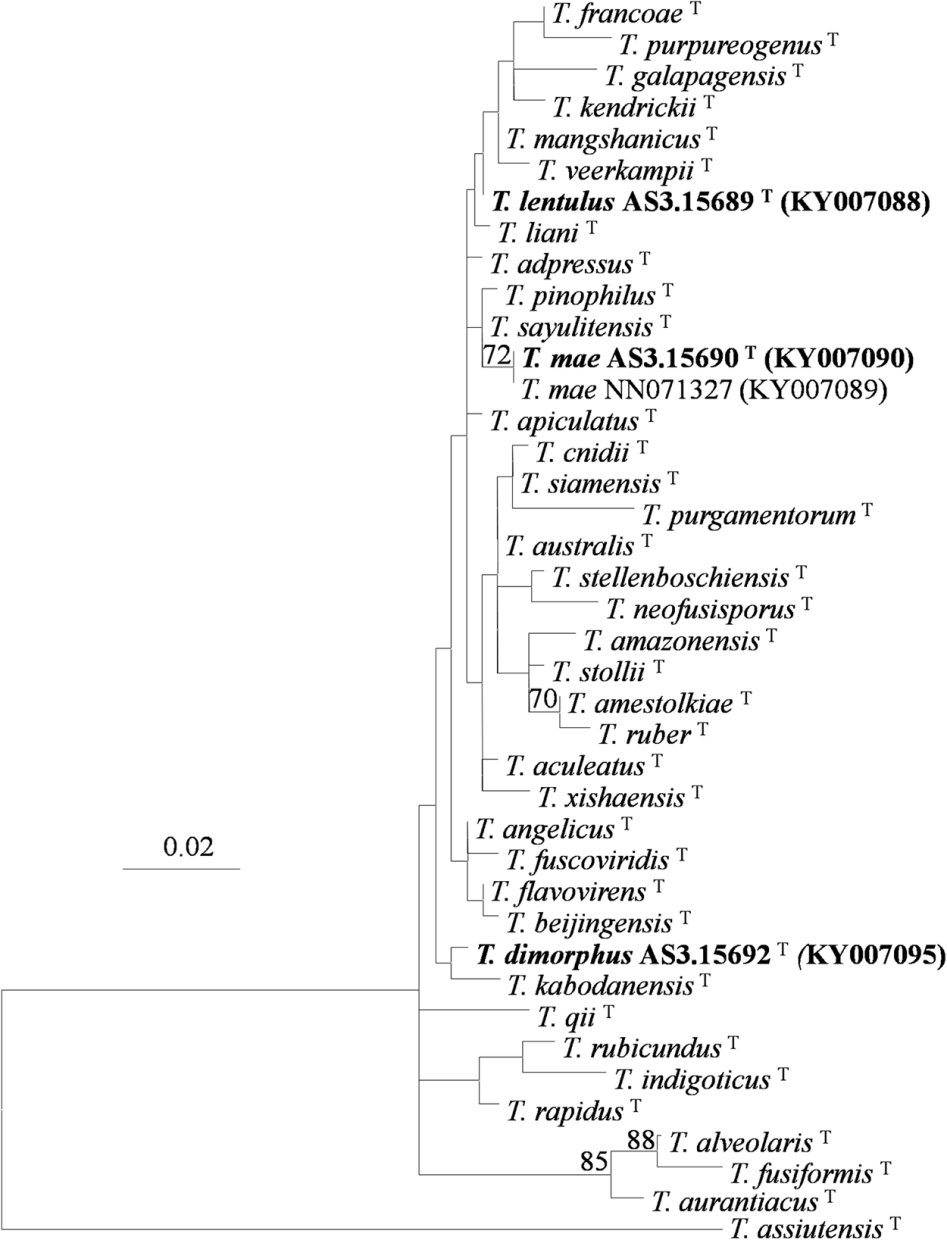
ML phylogram inferred from partial *ITS* sequences. Bootstrap percentages over 70% derived from 1000 replicates are indicated at the nodes. Bar = 0.05 substitutions per nucleotide position.

The phylogenetic trees generated by either the four individual loci or the concatenated *CaM*-*BenA-ITS* sequences show four isolates as three distinct, monophyletic species (Figs 1–3, S1–3).These phylograms all present that the three new members, i. e. *T*. *dimorphorus*, *T*. *lentulus* and *T*. *mae* belong to Sect. *Talaromyces*. *T*. *dimorphorus* is so distinctive that no close relatives are found in the section. *T*. *adpressus*, *T*. *lentulus*, *T*. *mae* and *T*. *pinophilus* are grouped in one clade with 100%, 99% and 99% bootstrap support according to *CaM*, *Rpb2* and *CaM*-*BenA-ITS* sequences, respectively. But the phylogram resulted from *BenA* indicates that *T*. *adpressus*, *T*. *lentulus*, and *T*. *sayulitensis* are closely related with a bootstrap support of 80% while *T*. *mae* is in an outgroup clade to them, however, the *ITS* phylogram does not show that these species are related.

**Figure 4:**
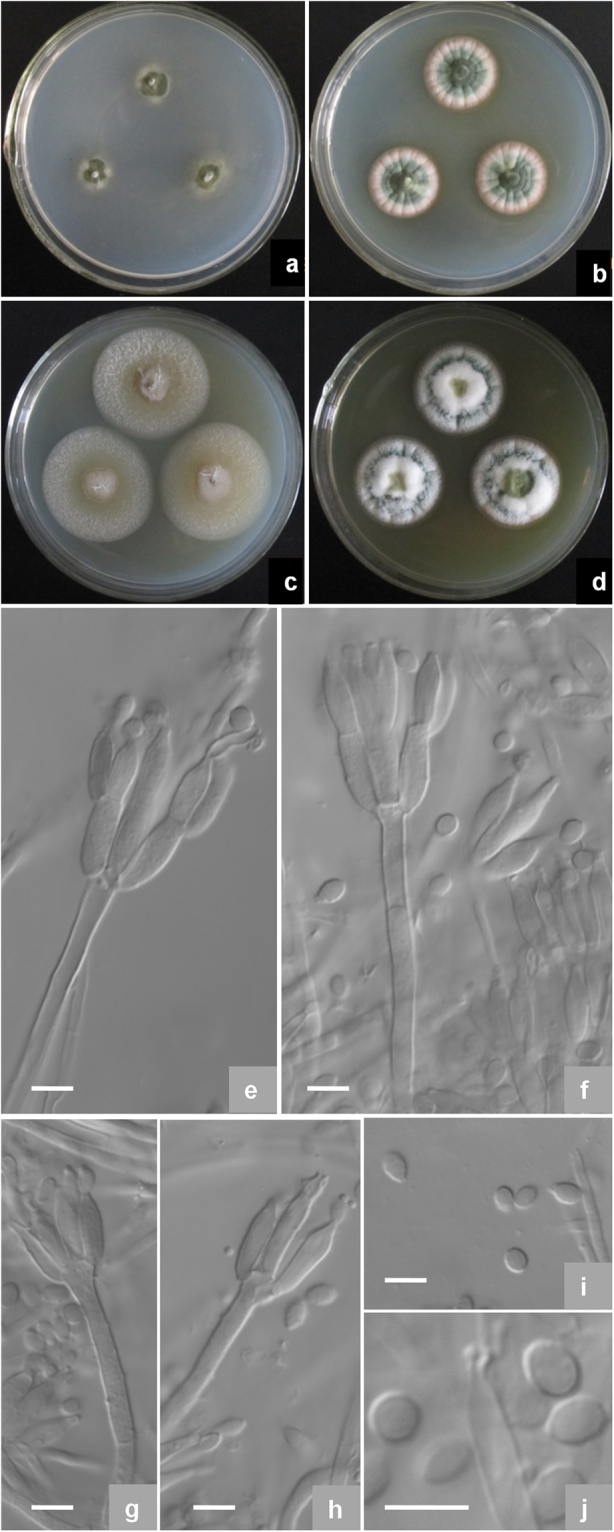
Morphological characters of *T*. *dimorphus* AS3.15692 ^T^ incubated at 25 °C for 7 days. (**a**) Cz; (**b**) CYA; (**c**) MEA; (**d**) YES; (**e**–**h**) Conidiophores; (**i**–**j**) Conidia. Bar = 5 µm.

**Description of** ***Talaromyces dimorphus*** X.-Z. Jiang & L. Wang, sp. nov., Fig. 4

Fungal Names: FN 570521; MycoBank: MB 824518

Etymology: The specific epithet is derived from that both biverticillate and monoverticillate penicilli are commonly produced by the species.

Holotype: HMAS 247023

Colonies 16–18 mm diam on **Cz** at 25 °C after 7 d, thin, plane, margins submerged, irregular; velutinous; conidiogenesis moderate, near Grass Green (R. Pl. VI); mycelium white; no exudate and soluble pigment; reverse Water Green (R. Pl. XLI). Colonies 23–25 mm diam on **CYA** at 25 °C after 7 d, thin, radially sulcate; velutinous; conidiogenesis moderate, in central areas, coloured Pistachio Green to Leaf Green (R. Pl. XLI); mycelium white; no exudates and soluble pigment; reverse Reed Yellow to Olive Yellow (R. Pl. XXX). Colonies 38–39 mm diam on **MEA** at 25 °C after 7 d, low, plane; slimy texture overlaid with sparse, short and white mycelial funicles about 1–3 mm, and longer in centres; conidiogenesis absent; no exudate or soluble pigment; reverse Cream Color (R. Pl. XVI) and near Buckthorn Brown centrally. Colonies 29–30 mm diam on **YES** at 25 °C after 7 d, thin, radially sulcate; funiculose, but floccose centrally; conidiogenesis limited, Niagara Green (R. Pl. XXXIII); mycelium white; exudate and soluble pigment absent; reverse Buckthorn Brown (R. Pl. XV). On CYA at **37 °C** after 7 d, no growth. On CYA at **5 °C** after 7 d, no growth.

Conidiophores arising from funicles and surface hyphae; stipes (15–) 25–50 (−70) × 2.5–3.5 μm, smooth-walled; penicilli biverticillate and monoverticillate; metulae (2–) 4–6 per vertical, 9–13 × 2.5–3.5 μm; phialides 2–4 per verticil, ampulliform, 7–11 × 2.5–3.0 μm, with short and blunt collula; conidia ovoid to ellipsoidal, 2.5–3.5 μm, smooth-walled, borne in short divergent chains about 45–60 μm long.

*Strains examined*: China, Hainan, Jianfengling Forest Park, 18°43′12″N 108°49′48″E, 1300 m, from soil, 8 Nov. 2015, coll. X.-Z. Jiang, ex-type culture AS3.15692 = NN072337 (Holotype: HMAS 247023, from dried culture of ex-type AS3.15692 on CYA).

*Notes:* This new taxon is characterized by sparse slimy colonies on MEA, short stipes, biverticillate and monoverticillate penicilli, ampuliform phialides with short blunt collula and smooth-walled conidia.

**Figure 5:**
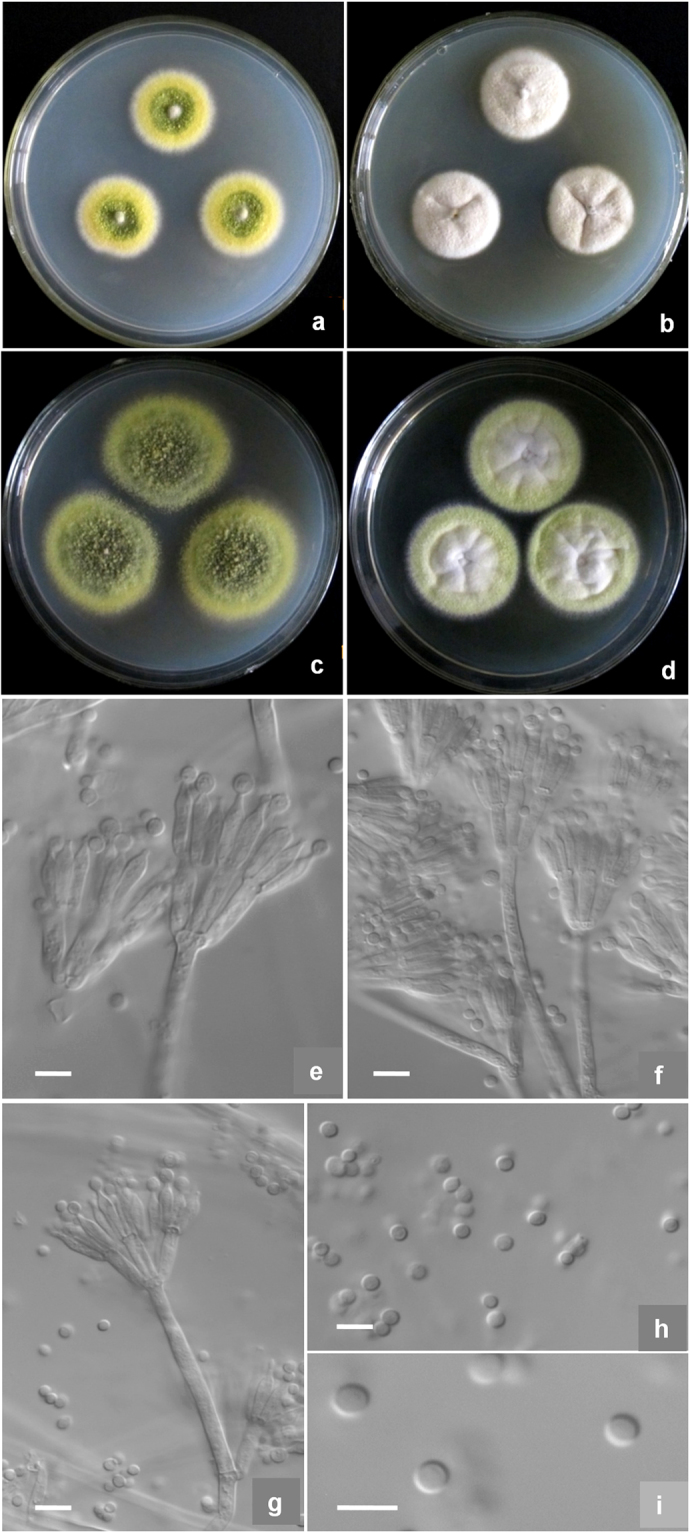
Morphological characters of *T*. *lentulus* AS3.15689 ^T^ incubated at 25 °C for 7 days. (**a**) Cz; (**b**) CYA; (**c**) MEA; (**d**) YES; (**e**–**g**) Conidiophores; (**h**–**i**) Conidia. Bar = 5 µm.

**Description of** ***Talaromyces lentulus*** X.-Z. Jiang & L. Wang, sp. nov., Fig. 5

Fungal Names: FN 570522; MycoBank: MB 824519

Etymology: The specific epithet is derived from its late development of conidiogenesis on CYA and YES.

Holotype: HMAS 247024

Colonies 26–28 mm diam on **Cz** at 25 °C after 7 d, thin, plane, umbonate in centers; velutinous; conidiogenesis limited to moderate in central areas, near Spinach Green (R. Pl. V); mycelium Green Yellow (R. Pl. V); no exudate and soluble pigment; reverse Light Buff (R. Pl. XV), slightly with variegated Flesh Color (R. Pl. XIV). Colonies 26–27 mm diam on **CYA** at 25 °C after 7 d, thin, with few radial sulci; velutinous with sparsely overlaid mycelium; conidiogenesis absent, mycelium near Pale Salmon Color (R. Pl. XIV), slightly mingled with Naphthalene Yellow (R. Pl. XVI); exudate absent or limited, clear; no soluble pigment; reverse Cinnamon (R. Pl. XXIX). Colonies 43–44 mm diam on **MEA** at 25 °C after 7 d, moderately deep, plane; velutinous with sparse floccose mycelium overlaid; conidiogenesis moderate, near Grayish Olive to Light Grayish Olive (R. Pl. XLVI); mycelium Light Viridine Yellow (R. Pl. V); no exudate and soluble pigment; reverse Baryta Yellow (R. Pl. IV). Colonies 37–38 mm diam on **YES** at 25 °C after 7 d, slightly deep, irregularly plicate in centres; velutinous, and floccose with short loose funicles in centres; conidiogenesis sparse; mycelium Citron Yellow (R. Pl. XVI), Pale Pinkish Buff (R. Pl. XVI) in central areas; exudate and soluble pigment absent; reverse Mahogany Red to Burnt Sienna (R. Pl. II).On CYA at 37 °C after 7 d, colonies 18–21 mm diam, thin, plane; velutinous; no conidiogenesis, mycelium coloured Pale Salmon Color (R. Pl. XIV); exudate and soluble pigment; reverse Antique centrally and Pale Yellow-Orange in other areas (R. Pl. III). On CYA at 5 °C after 7 d, no growth.

Conidiophores arising from surface hyphae; stipes 240–380 × 2.5–3.0 μm, smooth-walled; penicilli biverticillate; metulae 4–6 per stipe, 10–11 × 2.0–2.5 μm; phialides 2–4 per metula, acerose with short collula, 9–10 × 1.5–2.0 μm; conidia globose, 2.5–3.0 μm, smooth-walled, conidial chains irregularly tangled into loose massed, about 40–60 μm long.

*Strains examined*: China, Shandong, Dongying, 37°43′12N 118°47′24E, 8 m, from soil, 15 Sep. 2015, coll. X.-Z. Jiang, ex-type culture AS3.15689* = *NN071323 (Holotype: HMAS 247024 from dried culture of ex-type AS3.15689 on CYA).

*Notes:* This new species is characterized by vivid yellow mycelium, late development of conidiogenesis on CYA and YES, and good growth at 37 °C.

**Figure 6:**
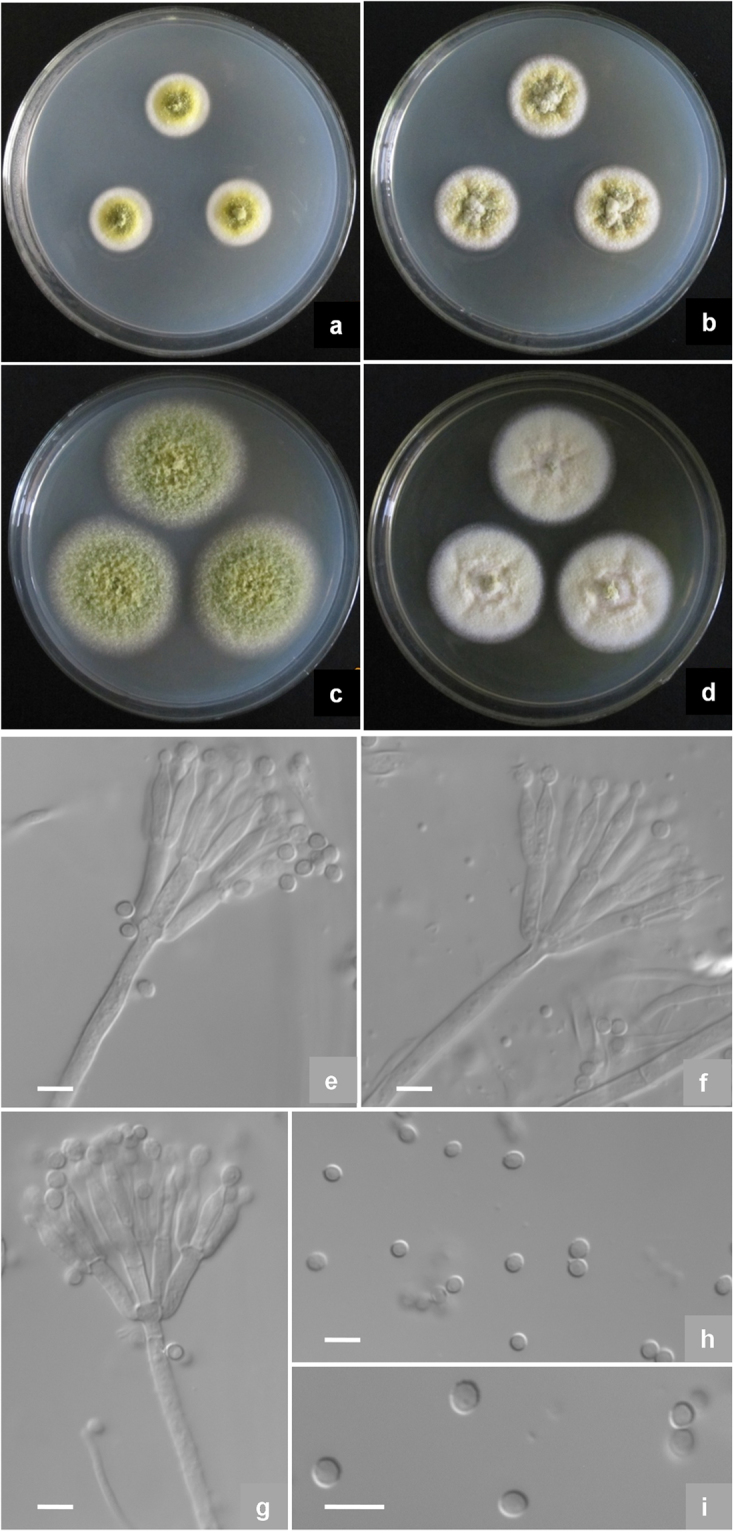
Morphological characters of *T*. *maes* AS3.15690 ^T^ incubated at 25 °C for 7 days. (**a**) Cz; (**b**) CYA; (**c**) MEA; (**d**) YES; (**e**–**g**) Conidiophores; (**h**–**i**) Conidia. Bar = 5 µm.

**Description of** ***Talaromyces mae*** X.-Z. Jiang & L. Wang, sp. nov., Fig. 6

Fungal Names: FN 570523; MycoBank: MB 824520

Etymology: named after Mrs. Xin-Yi Ma, who is the first scholar reporting *Aspergillus* and *Penicillium* species in China in the year of 1936.

Holotype: HMAS 247025

Colonies 18–19 mm diam on **Cz** at 25 °C after 7 d, thin, plane, slightly protuberate centrally; velutinous; conidiogenesis limited, near Serpentine Green (R. Pl. XVI); mycelium white at margins but Strontian Yellow in central areas (R. Pl. XLVI); no exudate and soluble pigment; reverse Cream Color (R. Pl. XVI). Colonies 22–24 mm diam on **CYA** at 25 °C after 7 d, thin, irregularly sulcate; velutinous with sparsely overlaid mycelium; conidiogenesis limited, in central areas, near Serpentine Green (R. Pl. XVI); mycelium white at margins while Straw Yellow (R. Pl. XVI) in other areas; clear exudate limited, no soluble pigment; reverse Baryta Yellow (R. Pl. IV) and with Salmon Color (R. Pl. XIV) centrally. Colonies 42–43 mm diam on **MEA** at 25 °C after 7 d, slightly deep, plane; funiculose and floccose with funicles about 1–3 mm; conidiogenesis sparse, near Light Elm Green (R. Pl. XVII); mycelium Light Dull Green-Yellow (R. Pl. XVII); no exudate and soluble pigment; reverse Cream Color (R. Pl. XVI). Colonies 33–34 mm diam on **YES** at 25 °C after 7 d, thin, slightly irregularly plicate; funiculose and floccose; conidiogenesis absent; mycelium Ivory Yellow to Primrose Yellow (R. Pl. XXX); exudates and soluble pigment absent; reverse Wax Yellow (R. Pl. XVI). On CYA at 37 °C after 7 d, colonies 17–18 mm diam, plane, slightly deep; velutinous, no conidiogenesis, exudate and soluble pigment. On CYA at 5 °C after 7 d, no growth.

Conidiophores arising from aerial hyphae and hyphal funicles; stipes (50–) 60–100 × 2.5–3.0 μm, smooth-walled; penicilli biverticillate; metulae 4–6 per stipe, 8–10 × 2.0–2.5 μm; phialides 2–4 per metula, acerose with short collula, 8–10 × 1.5–2.0 μm; conidia ovoid, 2.0–2.5 μm, walls smooth to finely rough, born in short irregularly tangled chains about 40–60 μm long.

*Strains examined*: China, Shanghai, Dongping Forest Park, 31°40′48″N 121°28′48″E, 3.5 m, from soil, 20 Sep. 2015, coll. X.-Z. Jiang, ex-type culture AS3.15690 = NN071328 (Holotype: HMAS 247025, from dried culture of ex-type AS3.15690 on CYA). Shandong: Dongying, 37°43′12″N 118°45′10″E, 8 m, from soil, 15 Sep. 2015, coll. X.-Z. Jiang, additional culture NN071327.

*Notes:* This new taxon is characterized by vivid yellow mycelium and hyphal funicles on MEA, good growth at 37 °C, and ovoid smooth-walled conidia.

## Discussion

Sect. *Talaromyces* is the largest section of the genus *Talaromyces*, and of which many new species were discovered following the monographic study of Yilmaz *et al*.^13^, which now include 52 species until the publication of Guevara-Suarez^24^. Apart from the teleomorphs, the members in this section show a great diversity in morphological characters. For example, on CYA at 25 **°**C some species grow very fast such as *T*. *rapidus* (44–46 mm) while some ones grow considerable slowly such as *T*. *mangshanicus* (6–7 mm). The colony texture is also varied greatly from strict velutinous (e. g. *T*. *qii*) to floccose (e. g. *T*. *derxii*) and even synnematous (e. g. *T*. *duclauxii*) or funiculous (e. g. *T*. *funiculosus*). As for microscopic characters, some members bear typical compact biverticillate penicilli (e. g. *T*. *beijingensis*), whereas, some species produce divergent ones (e. g. *T*. *flavovirens*). In addition, though the majority typically have biverticillate penicilli, certain species commonly bear both biverticillate and monoverticillate ones, such as *T*. *liani*. Moreover, most of the members produce acerose phialides, while some bear typical ampuliform ones, e. g. *T*. *stellenboschensis* and *T*. *mangshanicus*.

One new member reported here, namely *T*. *dimorphus* commonly shows both bivertillate and monoverticillate penicilli with ampuliform phialides, and moderate growth on CYA at 25 °C. These characters much resemble those of *T*. *veerkampii*, whereas, the striking differences between them lie in the colony characters. For instance, *T*. *veerkampii* shows sparse sporulation on CYA at 25 °C, and dense sporulation on MEA, fast growth on YES (35–46 mm) with bronze-green colony reverse, while *T*. *dimorphus* produces moderate conidia on CYA at 25 °C, slimy and sparse colony texture on MEA overlaid by mycelial funicles, moderate growth on YES (29–30 mm) with brown reverse colour. Moreover, *T*. *veerkampii* grows well at 37 °C (18–23 mm)^14^, but *T*. *dimorphus* presents no growth at this temperature. In addition, the flask-shaped phialides of *T*. *veerkampii* bears gradually tapered thin collula, and the conidial walls are finely rough, while the new species produces phialides with short blunt necks, and its conidia are smooth-walled. The molecular evidence of the phylograms based on *CaM*, *BenA*, *ITS* and *Rpb2* as well as the concatenated *CaM*-*BenA-ITS* sequences all shows that *T*. *dimorphus* is such a distinct species that no close relatives are found hitherto (Figs 1–3, S1–3).

In the phylograms according to *CaM*, *Rpb2* and *CaM*-*BenA-ITS* sequences, *T*. *adpressus*, *T*. *lentulus*, *T*. *mae* and *T*. *pinophilus* are in one clade with strong bootstrap support (100%, 99% and 99%, respectively, Figs 1, S1–2), and the phylogram resulted from *BenA* shows that *T*. *lentulus*, *T*. *adpressus*, and *T*. *sayulitensis* are in one clade with a bootstrap support of 80%, while *T*. *mae* and *T*. *pinophilus* are in an outer clade to these 3 species (Fig. 2, S3). Moreover, the *ITS* tree does not show these 5 species are close-related (Fig. 3). Morphological resemblance is in accordance with the relationship among these closely related species shown by molecular evidence. Visagie *et al*.^14^ and Chen *et al*.^21^ reported *T*. *sayulitensis* and *T*. *adpressus* respectively, which are morphologically very similar to *T*. *pinophilus*. The two new members, *T*. *lentulus* and *T*. *mae* are hardly distinguished from *T*. *pinophilus* by morphological characters too. The most remarkable morphological difference between *T*. *lentulus* and *T*. *pinophilus* is the length of stipes, which in *T*. *pinophilus*, is much shorter (30–200 μm) than that of *T*. *lentulus* (240–380 μm). Moreover, the two species are well-distinguished by all the phylograms (Figs 1–3, S1–3). Further, when 5 additional *T*. *pinophilus* isolates apart from the ex-type isolate CBS 631.66 were included in *BenA* analysis, *T*. *pinophilus* and *T*. *lentulus* are well separated (Fig. S3), which presents that *T*. *lentulus* is not a distinctive isolate of *T*. *pinophilus*. *T*. *lentulus* can be distinguished from *T*. *sayulitensis* in that the new species shows moderate conidiogenesis on MEA, while *T*. *sayulitensis* hardly produces conidia on MEA, and *T*. *lentulus* grows more slowly (18–21 mm) than *T*. *sayulitensis* (32–40 mm) at 37 °C. Still, *T*. *lentulus* seldom bears penicilli with sub-terminal branches, whereas sub-terminal branches are sometimes found in *T*. *sayulitensis*. Again, the two species can be well distinguished phylogeneically (Figs 1–3, S1–3). The differences between *T*. *lentulus* and *T*. *adpressus* are ready. For instance, *T*. *lentulus* produces yellow mycelium on MEA and YES, but *T*. *adpressus* bears white mycelium on these culture media. Microscopically, *T*. *lentulus* has much longer and thinner stipes (240–380 × 2.5–3.0 μm) than *T*. *adpressus* does (100–200 × 3–4.5 μm).

The differences between the two new members, *T*. *mae* and *T*. *lentulus* are even subtle. In general, *T*. *mae* grows somewhat slowly than *T*. *lentulus* (Cz 18–19 mm; CYA 22–24 mm; 42–43 mm; YES 33–34; CYA 37 °C 17–18 mm vs. Cz 26–28 mm; CYA 26–27 mm; 43–44 mm; YES 37–38; CYA 37 °C 18–21 mm), and the most notable difference is that *T*. *mae* shows a funiculous texture on MEA but *T*. *lentulus* does not. Moreover, *T*. *mae* bears conidiophores on aerial and funiculous hyphae, and accordingly shorter stipes (60–100 μm) while *T*. *lentulus* produces conidiophores on surface hyphae with longer stipes (240–380 μm). More important, the molecular evidence unequivocally shows them as different species (Figs 1–3, S1–3), and *T*. *mae* is not a strain of *T*. *pinophilus* either (Fig. S3).

Although establishing new species based on one single isolate or specimen may mistake certain isolates (or populations) of a known species for novel species, this can be avoided by examining the clade containing the studied isolates and the isolates of a known species in the phylograms of different individual genes. Empirically, if the studied isolates in the same clade with the isolates of a known species all have very few substitutions (very short or no branch length in phylograms) from the nearest node in every gene tree and with a strong bootstrap support (over 80%), then the studied isolates can be regarded as the known species, such as the clade including six isolates of *T*. *pinophilus* in Fig. S3 or the *T*. *ruglosus* isolates in the study of Yilmaz *et al.*^15^. In this study, the two isolates AS3.15689 and AS3.15692 of the proposed new taxa *T*. *lentulus* and *T*. *dimorphus*, respectively, are well separated from other isolates of known species in all the trees, thus, their novelty status can be verified.

When we inferring the phylogenetic relationships using concatenated sequences of different genes, there is an assumption that all these genes have undergone the same evolutionary ways, but in nature, it is not the case. On the whole, different genes have different evolutionary ways, thus have different mutation models, so we preferred drawing phylograms based on individual gene sequences, which may result in incongruent relationships among certain species, though. For example, *T*. *francoae*, *T*. *kendrickii*, *T*. *mangshanicus* and *T*. *qii* and are closely related in *CaM* and *BenA* trees (Figs 1,2, S3), but in *ITS* tree, *T*. *mangshanicus* has no close relatives (Fig. 3), while in *rpb2* tree *T*. *aculeatus* and *T*. *mangshanicus* are siblings (Fig. S1). When the ex-type *Rpb2* sequences of *T*. *aculeatus* (KM023287) and *T*. *mangshanicus* (KX447527) are compared with each other, only 3 nucleotides are found different in the alignment of 852 nucleotides. The explanation may be that *Rpb2* gene of these two species evolved in the same way, which is different from *CaM*, *BenA* and *ITS*. For another instance, *T*. *aculeatus* and *T*. *apiculatus* are close-related in *CaM* and *BenA* phylograms (Figs 1,2, S3), whereas, they are well separated in *ITS* and *Rpb2* trees (Figs 3, S1). Thus, it seems normal that *T*. *sayulitensis* lies in the outgroup position to *T*. *adpressus* and *T*. *lentulus* in *CaM* tree (Fig. 1), but is closely related to *T*. *adpressus* and *T*. *lentulus *in *BenA* trees (Figs 2, S3). However, the 3 species *T*. *adpressus*, *T*. *lentulus* and *T*. *sayulitensis* are all separated in the *ITS* phylogram (Fig. 3). Notwithstanding this incongruence, the novelty status of the *T*. *dimorphus*, *T*. *lentulus* and *T*. *mae* can be verified by all the four individual gene trees.

## Materials and Methods

### Isolation of strains

Soil samples were collected and kept in sterilized plastic bags. The dilution plating method was used in the isolation of the fungi^26^. The strains of *Talaromyces* were deposited in Novozymes Culture Collection (Novozymes (China) Investment Co. Ltd., Beijing 100085, China) as NN072337, NN071323, NN071328 and NN071327, and the 3 ex-type cultures of *T*. *dimorphus*, *T*. *lentulus* and *T*. *mae* were also deposited in China General Microbiological Culture Collection (CGMCC) as AS3.15692 = NN072337, AS3.15689 = NN071323, AS3.15690 = NN071328, respectively. The dried specimens of the holotypes from the ex-type cultures were deposited in the Herbarium Mycologicum Academiae Sinicae as HMAS 247023, HMAS 247024, HMAS 247025, respectively.

### Morphological studies

Colony characters were assessed using Czapek agar (Cz)^2^, Czapek yeast autolysate agar (CYA)^4^, 2% malt extract agar (MEA, malt extract (Difco, Lawrence, Kansas, USA)^4^, YES (yeast extract sucrose agar (Oxoid, Basingstoke, Hants, UK)^27^. Colour names followed those of Ridgway^28^. Microscopic mounts were prepared using material from colonies growing on MEA at 25 °C after 7 days mounted in 90% lactic acid without dye. Microscopic examination and photography were performed with an Axioplan2 imaging and Axiophot2 universal Microscope (Carl Zeiss (Shanghai) Co. Ltd., Shanghai, China).

### Molecular studies

DNA extraction followed the method of Scott *et al*.^29^. Partial calmodulin gene (*CaM*) was amplified using the primers cmdAD1 and cmdQ1^30^; partial β-tubulin gene (*BenA*) sequences were obtained with Bt2a and Bt2b^31^; the ITS1-5.8S-ITS2 region (*ITS*) of nuc rDNA was amplified using ITS5 and ITS4^32^, and the partial DNA-dependent RNA polymerase II second largest subunit gene (*Rpb2*) sequences were obtained with sense primers rpb2T1: 5′-act ggt aac tgg ggt gag ca-3′or T2: 5′-acg ggt aac tgg ggt gaa ca-3′ with antisense primers rpb2E1: 5′-tc aca gtg agt cca ggt gtg-3′or E2: 5′-tc gca atg cgt cca ggt atg-3′. Polymerase chain reactions (PCR) were carried out in 20 µL reaction mixture containing 0.5 µL of each primer (10 pmol/µL), 1.0 µL of genomic DNA (10 ng/µL), 10 µL of 2 × PCR MasterMix buffer (0.05 u/µL Taq polymerase, 4 mM MgCl_2_, 0.4 mM dNTPs), and 8 µL of double distilled water (Tsingke Co. Ltd, Beijng, China). Amplifications were performed in a PTC-200 thermocycler (MJ Research, Watertown, Massachusetts, USA), the reaction program consisted of 94 °C for 3 min; 94 °C for 30 s, 50 °C for 30 s, 72 °C for 30 s, 34 cycles; 72 °C for 5 min. After amplification the PCR amplicons were electrophoresed in a 2.0% agarose gel soaked in TAE buffer with a 100 bp DNA ladder (MBI Fermentas, Burlington, Ontario, CA) at 100 V for 15 min. The gel were then stained in an aqueous 0.5 μg/mL ethidium bromide water solution for 10 min and examined under 254 nm UV using a portable UV light in a dark room. Samples showing one single, obvious band of the anticipated length in the gel were then purified and sequenced on both strands with an ABI 3730 DNA analyzer (Applied Biosystems, Waltham, Massachusetts, USA). Raw sequences were proof-read and edited manually with BioEdit 7.0.9^33^. Edited sequences were aligned using muscle in MEGA version 6^34^. Forty strains of *Talaromyces* were included in *CaM*, *BenA*, *ITS* and the concatenated *CaM*-*BenA-ITS* phylogenetic analyses with sequences from ex-types. Only 18 sequences were obtained in *Rpb2* analysis. The five sequence matrices were analyzed using Maximum Likelihood (ML) method and subjected to 1000 bootstrap replications, with substitution model and rates among sites K2+G+I for *CaM*, K2+G for *BenA*, *Rpb2* and *CaM*-*BenA-ITS*, and T92+G+I for *ITS*. Gaps were treated as partial deletion according to Hall^35^. *T*. *assiutensis* CBS147.78 ^T^ was chosen as the outgroup (Table 1).

**Table 1:** Forty strains included in phylogenetic analyses with *T*. *assiutensis* as the outgroup, and the GenBank accession numbers of four genetic markers.

Species	Strains^a^	Source	Genetic markers^b^			
			*CaM*	*BenA*	*ITS*	*Rpb2*
*T*. *aculeatus*	CBS 289.48 ^T^	Textile, USA	KF741975	KF741929	KF741995	KM023271
*T*. *adpressus*	CGMCC 3.18211 ^T^	Indoor air, Beijing, China	KU866741	KU866844	KU866657	KU867001
*T*. *alveolaris*	CBS 142379 ^T^	Human bronchoaveolar lavage, Utah, USA	LT795596	LT559086	LT558969	N/A
*T*. *amazonensis*	CBS 140373 ^T^	Leaf litter from 6-month old litterbag in mature forest in Araracuara, dept. Amazonas, Colombia	KX011502	KX011490	KX011509	N/A
*T*. *amestolkiae*	CBS 132696 ^T^	House dust, South Africa	KF741937	JX315623	JX315660	JX315698
*T*. *angelicus*	KACC 46611 ^T^	Dried roots of *Angelica gigas*, Pyeongchang, Korea	KJ885259	KF183640	KF183638	N/A
*T*. *apiculatus*	CBS 312.59 ^T^	Soil, Japan	KF741950	KF741916	JN899375	KM023287
*T*. *assiutensis*	CBS 147.78 ^T^	Soil, Egypt	KJ885260	KJ865720	JN899323	N/A
*T*. *aurantiacus*	CBS314.59 ^T^	Soil, Georgia, USA	KF741951	KF741917	JN899380	N/A
*T*. *australis*	CBS 136667 ^T^	Aerial contaminant, Rydalmere,New South Wales, Australia	KF741969	KF741923	KF741989	N/A
*T*. *beijingensis*	CGMCC 3.18200 ^T^	Indoor air, Beijing, China	KU866733	KU866837	KU866649	KU866993
*T*. *cnidii*	KACC 46617 ^T^	Dried roots of *Cnidium officinale*, Chungbuk, Republic of Korea	KJ885266	KF183641	KF183639	KM023299
***T***. ***dimorphus*** **X**.**-Z**. **Jiang & L**. **Wang**	**AS3**.**15692 **^T^** = NN072337**	**Forest soil**, **Jianfengling Forest Park**, **Hainan**, **China**	**KY007103**	**KY007111**	**KY007095**	**KY112593**
*T*. *flavovirens*	CBS 102801 ^T^	Dead leaves of *Quercus ilex*, Parque del Retiro, Madrid, Spain	KF741933	JX091376	JN899392	N/A
*T*. *francoae*	CBS 113134 ^T^	Leaf litter from 4-month old litterbag in *Pseudomonotes tropenbosii* (Dipterocarpaceae) forest in Peña Roja, Dept. Amazonas, Colombia	KX011501	KX011489	KX011510	N/A
*T*. *fuscoviridis*	CBS 193.69 ^T^	Soil, the Netherlands	KF741942	KF741912	KF741979	N/A
*T*. *fusiformis*	CGMCC 3.18210 ^T^	Indoor air, Beijing, China	KU866740	KU866843	KU866656	KU867000
*T*. *galapagensis*	CBS 751.74 ^T^	Shaded soil under *Maytenusobovata*, Galapagos Islands, Isla Santa Cruz, Ecuador	KF741966	JX091388	JN899358	N/A
*T*. *indigoticus*	CBS 100534 ^T^	Soil, Minamikushiyama, Nagasaki, Japan	KF741931	JX494308	JN899331	N/A
*T*. *kabodanensis*	CBS 139564 ^T^	Hyper saline soil, Kabodan Island, Urmia Lake National Park, Iran	KP851995,	KP851986	KP851981	N/A
*T*. *kendrickii*	CBS 136666 ^T^	Forest soil, Canada	KF741967	KF741921	KF741987	N/A
***T***. ***lentulus*** **X**.**-Z**. **Jiang & L**. **Wang**	**AS3**.**15689** ^**T**^** = NN071323**	**Alkaline soil**, **Yingkou**, **Shandong**, **China**	**KY007096**	**KY007104**	**KY007088**	**KY112586**
*T*. *liani*	CBS 225.66 ^T^	Soil, China	KJ885257	JX091380	JN899395	N/A
***T***. ***mae*** **X**.**-Z Jiang & L**. **Wang**	**AS3**.**15690** ^**T**^** = NN071328**	**Forest soil**, **Chongming Island**, **Shanghai**, **China**	**KY007098**	**KY007106**	**KY007090**	**KY112588**
	NN071327	Alkaline soil, Yingkou, Shandong, China	KY007097	KY007105	KY007089	KY112587
*T*. *mangshanicus*	CGMCC 3.18013 ^T^	Soil, Mangshan National Nature Reserve, Hunan, China	KX447528	KX447530	KX447531	KX447527
*T*. *neofusisporus*	AS3.15415 ^T^	Plant leaves, Tibet, China	KP765383	KP765381	KP765385	N/A
*T*. *pinophilus*	CBS 631.66 ^T^	PVC, France	KF741964	JX091381	00634504 (NBRC 6345 ^c^)	KM023291
*T*. *purgamentorum*	CBS 113145 ^T^	Leaf litter from 4-month old litterbag in *Pseudomonotes tropenbosii* (Dipterocarpaceae) forest in Peña Roja, Dept. Amazonas, Colombia	KX011500	KX011487	KX011504	N/A
*T*. *purpurogenus*	CBS 286.36 ^T^	Parasitic on a culture of *Aspergillus oryzae*, Japan	KF741947	JX315639	JN899372	JX315709
*T*. *qii*	AS3.15414 ^T^	Leaves, Motuo, Tibet, China	KP765382	KP765380	KP765384	N/A
*T*. *rapidus*	CBS 142382 ^T^	Human bronchoaveolar lavage, Ohio, USA	LT795600	LT559087	LT558970	LT795601
*T*. *ruber*	CBS 132704 ^T^	Air craft fuel tank, United Kingdom	KF741938	JX315629	JX315662	JX315700
*T*. *rubicundus*	CBS 342.59 ^T^	Soil, Georgia	KF741956	JX494309	JN899384	N/A
*T*. *sayulitensis*	CBS 138204 ^T^	House dust, Mexico	KJ775422	KJ775206	KJ775713	N/A
*T*. *siamensis*	CBS 475.88 ^T^	Forest soil, Thailand	KF741960	JX091379	JN899385	KM023279
*T*. *stellenboschiensis*	CBS 135665 ^T^	Soil, Stellenbosch, South Africa	JX140683	JX091605	JX091471	N/A
*T*. *stollii*	CBS 408.93 ^T^	AIDS patient, the Netherlands	JX315646	JX315633	JX315674	JX315712
*T*. *veerkampii*	CBS 500.78 ^T^	Soil, Dep. de Meta, Municipio de Villavicencio, Columbia	KF741961	KF741918	KF741984	N/A
*T*. *xishaensis*	CGMCC 3.17995 ^T^	Xisha Islands, Sansha City, Hainan, China	KU644582	KU644581	KU644580	N/A

^a^AS, CGMCC, China General Microbiological Culture Collection, Academia Sinica, Beijing, China; CBS, Centraalbureau voor Schimmelcultures, Utrecht, the Netherlands; KACC, Korean Agricultural Culture Collection, Suwon, Republic of Korea; NN, Novozymes (China) Investment Co. Ltd, Beijing, China; ex-type strains are indicated with ^T^.

^b^Sequences KY007088−KY00711 and KY112586−KY112593 are obtained in this study.

^c^NBRC, NITE Biological Resource Center, Chiba, Japan.

## Electronic supplementary material

Figure S1–3
