# Supplementary material for: Three new species of Talaromyces sect. Talaromyces discovered from soil in China
Source: Sci Rep. 2018 Mar 21;8:4932. doi: 10.1038/s41598-018-23370-x (PMC5862941; doi:10.1038/s41598-018-23370-x)

Supplementary information of

Three new species of *Talaromyces* sect. *Talaromyces* discovered from soil in China

Xian-Zhi Jiang [#1], Zhong-Dong Yu [#2], Yong-Ming Ruan [3], Long Wang [4*]

[1] Novozymes (China) Investment Co. Ltd., Beijing 100085, China

[2] College of Forestry, Northwest A&F University, Yangling 712100, Shaanxi, China

[3] College of Chemistry and Life Sciences, Zhejiang Normal University, Jinhua 321004, Zhejiang, China

[4] State Key Laboratory of Mycology, Institute of Microbiology, Chinese Academy of Sciences, Beijing 100101, China

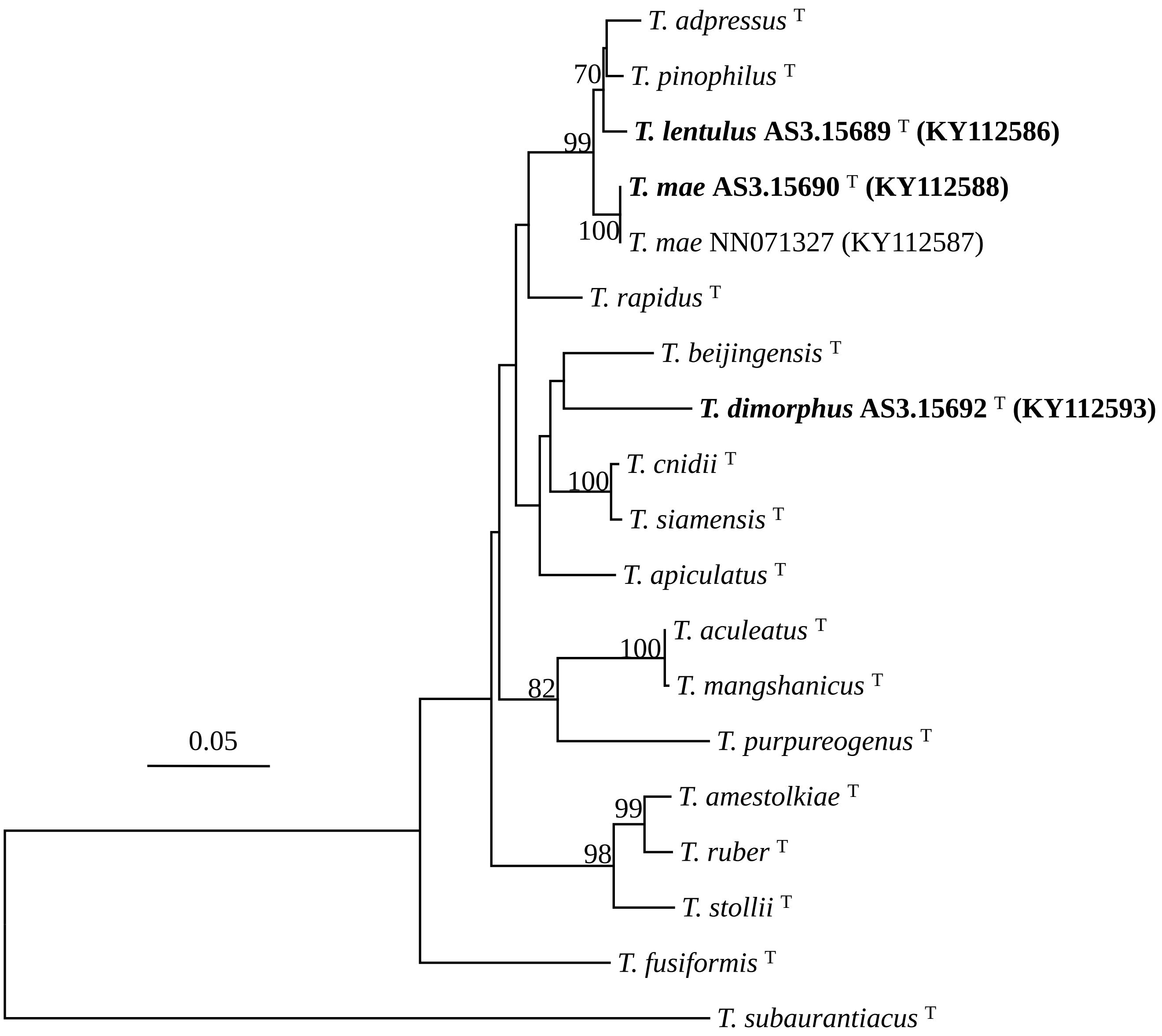

Fig. S1 ML phylogram inferred from partial *rpb2* sequences. Bootstrap percentages over 70% derived from 1000 replicates are indicated at the nodes. Bar = 0.05 substitutions per nucleotide position.

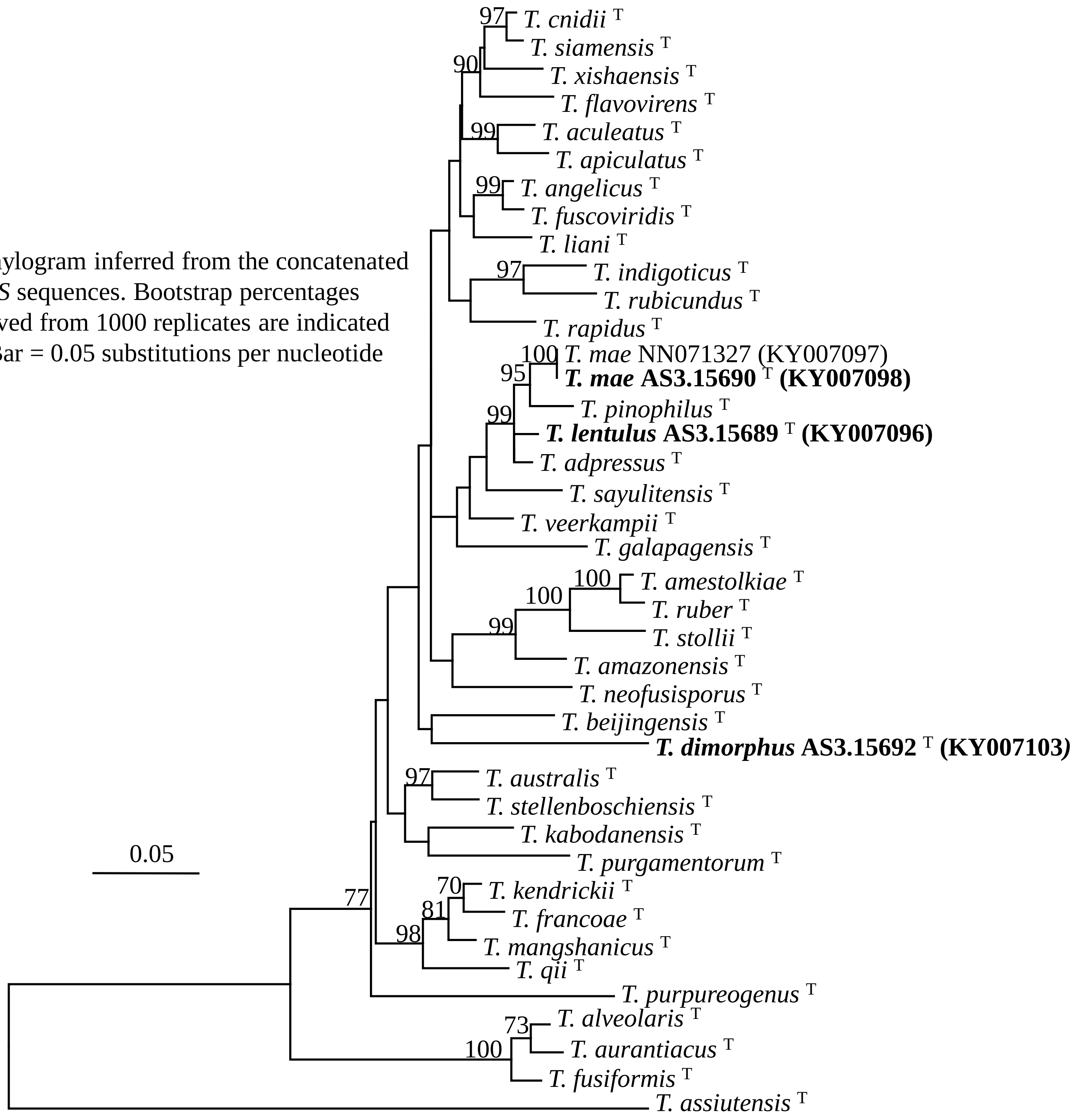

Fig. S2 ML phylogram inferred from the concatenated *CaM-BenA-ITS* sequences. Bootstrap percentages over 70% derived from 1000 replicates are indicated at the nodes. Bar = 0.05 substitutions per nucleotide position.

Fig. S3 ML phylogram inferred from partial *BenA* sequences with five additional isolates of *T. pinophilus*. Bootstrap percentages over 70% derived from 1000 replicates are indicated at the nodes. Bar = 0.05 substitutions per nucleotide position.

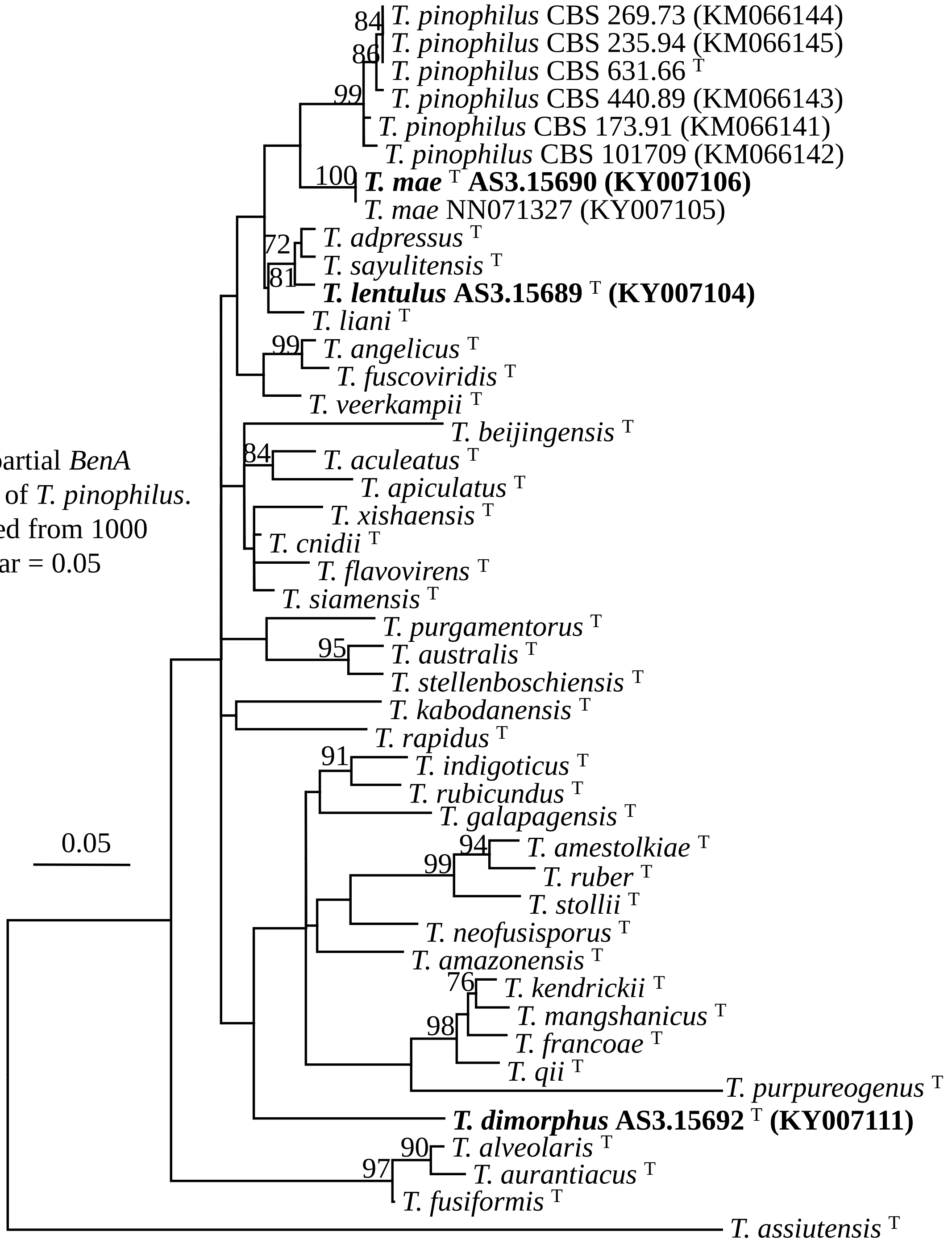

Supplement: Supplementary file 1 — Figure S1–3 [file 41598_2018_23370_MOESM1_ESM.pdf]
